# Assessment of changes in brain metabolites in Indian patients with type-2 diabetes mellitus using proton magnetic resonance spectroscopy

**DOI:** 10.1186/1756-0500-7-41

**Published:** 2014-01-17

**Authors:** Sanjeev Sinha, Meera Ekka, Uma Sharma, Raghunandan P, R M Pandey, N R Jagannathan

**Affiliations:** 1 Department of Medicine, All India Institute of Medical Sciences, Ansari Nagar, New Delhi 110029, India; 2 Department of NMR & MRI Facility, All India Institute of Medical Sciences, New Delhi, India; 3 Department of Biostatistics, All India Institute of Medical Sciences, New Delhi, India

**Keywords:** Diabetes mellitus, Brain metabolites, MR Spectroscopy

## Abstract

**Background:**

The brain is a target for diabetic end-organ damage, though the pathophysiology of diabetic encephalopathy is still not well understood. The aim of the present study was to investigate the effect of diabetes on the metabolic profile of brain of patients having diabetes in comparison to healthy controls, using *in*-*vivo* magnetic resonance spectroscopy to get an insight into the pathophysiology of cerebral damages caused due to diabetes.

**Methods:**

Single voxel proton magnetic resonance spectroscopy (^1^H-MRS) was performed at 1.5 T on right frontal, right parieto-temporal and right parieto-occipital white matter regions of the brain of 10 patients having type-2 diabetes along with 7 healthy controls. Absolute concentration of N-acetylaspartate (NAA), choline (cho), myo-inositol (mI), glutamate (Glu) and glutamine (Gln), creatine (Cr) and glucose were determined using the LC-Model and compared between the two groups.

**Results:**

The concentration of N-acetylaspartate was significantly lower in the right frontal [4.35 ±0.69 vs. 5.23 ±0.74; p = 0.03] and right parieto-occipital region [5.44 ±0.52 vs.6.08 ±0.25; p = 0.02] of the brain of diabetics as compared to the control group. The concentrations of glutamate and glutamine were found to be significantly higher in the right frontal region of the brain [7.98 ±2.57 vs. 5.32 ±1.43; P = 0.01] in diabetics. Glucose levels were found significantly elevated in all the three regions of the brain in diabetics as compared to the control group. However, no significant changes in levels of choline, myo-inositol and creatine were observed in the three regions of the brain examined among the two groups.

**Conclusions:**

^1^H-MRS analysis indicates that type-2 diabetes mellitus may cause subtle changes in the metabolic profile of the brain. Decreased concentrations of NAA might be indicative of decreased neuronal viability in diabetics while elevated concentrations of Gln and Glu might be related to the fluid imbalance resulting from disruption of glucose homeostasis.

## Background

Type-2 diabetes mellitus (DM) is one of the common metabolic diseases that affects approximately 61.3 million people in India alone. It has been projected to increase further to 101.2 million by 2030 [1]. DM is associated with cognitive deficits and an increased risk of dementia, particularly in the elderly [2]. A recent study reported that patients with type-2 DM show impairments in several cognitive domains, mostly in the area of verbal memory and processing speed [3]. Moreover, some recent evidences have shown that the brain is a target for diabetic end-organ damage, though the pathophysiology of diabetic encephalopathy is still not well understood. Both vascular and metabolic disturbances have been suggested to impair the integrity of the brain in diabetes [4]. Diffuse diabetic angiopathy, ischemic lesions and diffuse degenerative abnormalities have been described in patients with type-1 DM; however, in type-2 DM cognitive impairments may be relatively more pronounced that may be due to chronic hyperglycaemia, vascular disease, repeated hypoglycaemic episodes and possible direct effect of insulin on the brain as implicated in various studies [5]. A recent report documented the possible brain abnormalities caused among obese adolescents with type-2 DM; which may result from a combination of subtle vascular changes, glucose and lipid metabolic abnormalities, and subtle differences in adiposity in the absence of clinically significant vascular diseases [6].

Various imaging modalities including MRI (Magnetic Resonance Imaging), MRS (Magnetic Resonance Spectroscopy), CT scan, PET (Positron Emission Tomography), SPECT (Single-Photon Emission Computed Tomography) and cerebral blood flow imaging have been used to study the pathology of the brain inflicted by diabetes mellitus [7]. Proton magnetic resonance spectroscopy (^1^H-MRS) is a sensitive, non-invasive technique that provides metabolic information on the status of viability of neurons and on the membrane metabolism of the brain [8,9]. The brain metabolites, generally observed in ^1^H-MRS, are: N-acetylaspartate (NAA), choline (Cho), creatine (Cr), myo-inositol (mI), glutamate (Glu) and glutamine (Gln). Several researchers have studied the metabolic changes in the brain due to various clinical disorders affecting the central nervous system, including tumours, infarction and ischemia, multiple sclerosis, Alzheimer’s disease, and epilepsy [10,11]. It has also been used to assess metabolic changes in the brain of patients with obstructive sleep apnea (OSA) and COPD [12,13].

In the present study, a systematic investigation was carried out to understand the effect of diabetes on three regions of the brain of patients with type-2 DM using ^1^H-MRS. This study focused on the determination of absolute concentrations of NAA, Cho, Cr, Glx, mI and glucose in frontal, parieto-temporal and parieto-occipital white matter areas of the brain. This study was based on the hypothesis that cerebral metabolism is affected in type-2 diabetics due to chronic hyperglycaemia/hypoxia affecting the levels of cerebral metabolites. It was expected that patients with type-2 DM have lower levels of NAA, with concurrent higher levels of mI, cho and glucose.

## Methods

This prospective observational pilot study was conducted at All India Institute of Medical Sciences, New Delhi- a tertiary level referral centre. The protocol of the study was reviewed and approved by the ethics committee of All India Institute of Medical Sciences, New Delhi. All participants were informed about the study and their written consents were obtained.

### Selection of subjects

This study was conducted on ten patients with type 2 DM and seven healthy subjects who volunteered to participate in the study. The patients were recruited from the outpatient clinic of the Department of Medicine based on the following criteria: (1) age >30 years at the time of diagnosis of diabetes, (2) systolic blood pressure <130 mm Hg and diastolic blood pressure <80 mm Hg, (3) no proteinuria (urinary albumin excretion rate <20 μg/min, (4) no history of cerebrovascular and cardiovascular symptoms or disorders. The healthy subjects and patients were matched for age, gender, height and weight.

Complete history, physical examinations and laboratory tests were performed in all subjects to exclude the effect of diseases other than diabetes. All subjects had normal blood counts, serum creatinine levels, electrolyte concentrations and electrocardiograms. The subjects were not on antihypertensive, anticoagulants, or lipid-lowering drugs except for two cases. The patients were using oral hypoglycaemic agents for DM.

### Biochemical investigations

Venous blood samples were drawn after a 10–12 hour overnight fast for the estimation of blood glucose, total cholesterol (TC), serum triglyceride (TG), and high-density lipoprotein cholesterol (HDL-C). The total cholesterol, TG and HDL-C were measured using commercially available kits (Randox Laboratory, San Francisco, CA, USA) on a semi-automated analyser (Micro Semi-Autoanalyser 2000, C. L. Micromed, Italy). Low-density lipoprotein cholesterol (LDL-C) was estimated using Friedewald’s formula: LDL − C = TC − (HDL − C + TG/5) [14]. Non-HDL cholesterol was calculated by deducting the value of HDL − C from total cholesterol. Normal value for lipid was defined by the standard criteria used by National Cholesterol Education Program [15]. Diabetes mellitus was diagnosed according to the protocol of American Diabetes Association [16]. Glycosylated haemoglobin (HbA_1C_) assay was performed on HPLC (BioRad, Richmond, CA, USA).

### ^1^H-MRS of the brain

^1^H-MRS (proton magnetic resonance spectroscopy) analysis was performed on a total of 17 subjects (10 - diabetic and 7 - control) using volume-localized ^1^H-MRS at 1.5 Tesla MRI/MRS scanner (MAGNETOM, Siemens, Germany) using a circularly polarized (CP) coil. Prior to ^1^H-MRS analysis, multi-slice T1-weighted images of the coronal and sagittal planes of the whole brain were acquired using a standard spin-echo pulse sequence (TE = 15 ms; TR = 520 ms; 3–5 mm slice thickness; 256 × 256 matrix). T2-weighted axial images were acquired using the following parameters: TE = 90 ms; TR = 250 ms; 3–5 mm slice thickness; 256 ×2 56 matrix). These images were used to identify the region of interest for performing the volume-localized ^1^H-MRS. The spectra were recorded using PRESS pulse sequence with the following parameters: TR = 2000 ms; TE = 30 ms; NS = 128. Care was taken to optimise the magnetic field homogeneity by carrying out global and voxel shim. The line-width after voxel shim ranged from 5–9 Hz depending on the voxel size and the region studied. The spectra were acquired from three different regions of the brain viz. from right frontal, right parieto-temporal and right occipital white matter. However, spectra from all the three regions of the brain could not be recorded for all the subjects due to long acquisition time and patients’ non-cooperation. The spectra from right frontal white matter were acquired for all the ten patients and seven controls. While spectra from right parieto-temporal white matter were acquired for nine patients and five controls; that from the right occipital white matter were only acquired in eight diabetics and four controls.

### Statistical analysis

After confirming the approximate normality, normally distributed data were expressed as arithmetic means and standard deviation. The two groups were compared using Student’s *t*-test for continuous variables and x^2^ tests for categorical variables. Statistical analysis was performed using statistical software package ‘STATA version 10.0’ [(intercooled version), Stata Corporation, Houston, Texas, USA].

## Results

### Patient characteristics

Baseline blood chemistry reports were available only for eleven subjects as mentioned earlier. None of the healthy control subjects were smokers or alcoholics and have any history of hypertension, hyperlipidaemia or cerebrovascular disease. There was no differences among the individuals of the two groups with reference to age, sex, ethnicity, education, blood pressure level or BMI. As expected, with reference to the controls, the type-2 diabetic patients had significantly higher levels of fasting blood glucose, C-reactive protein and HbA_1C_ (*p* < 0.05). The groups did not show any significant differences with reference to the total cholesterol, LDL cholesterol, triglycerides and HDL cholesterol. The patients were diagnosed for type-2 DM 3.5 (±1.2) years earlier.

### Proton magnetic resonance spectroscopy

**Figure 1:**
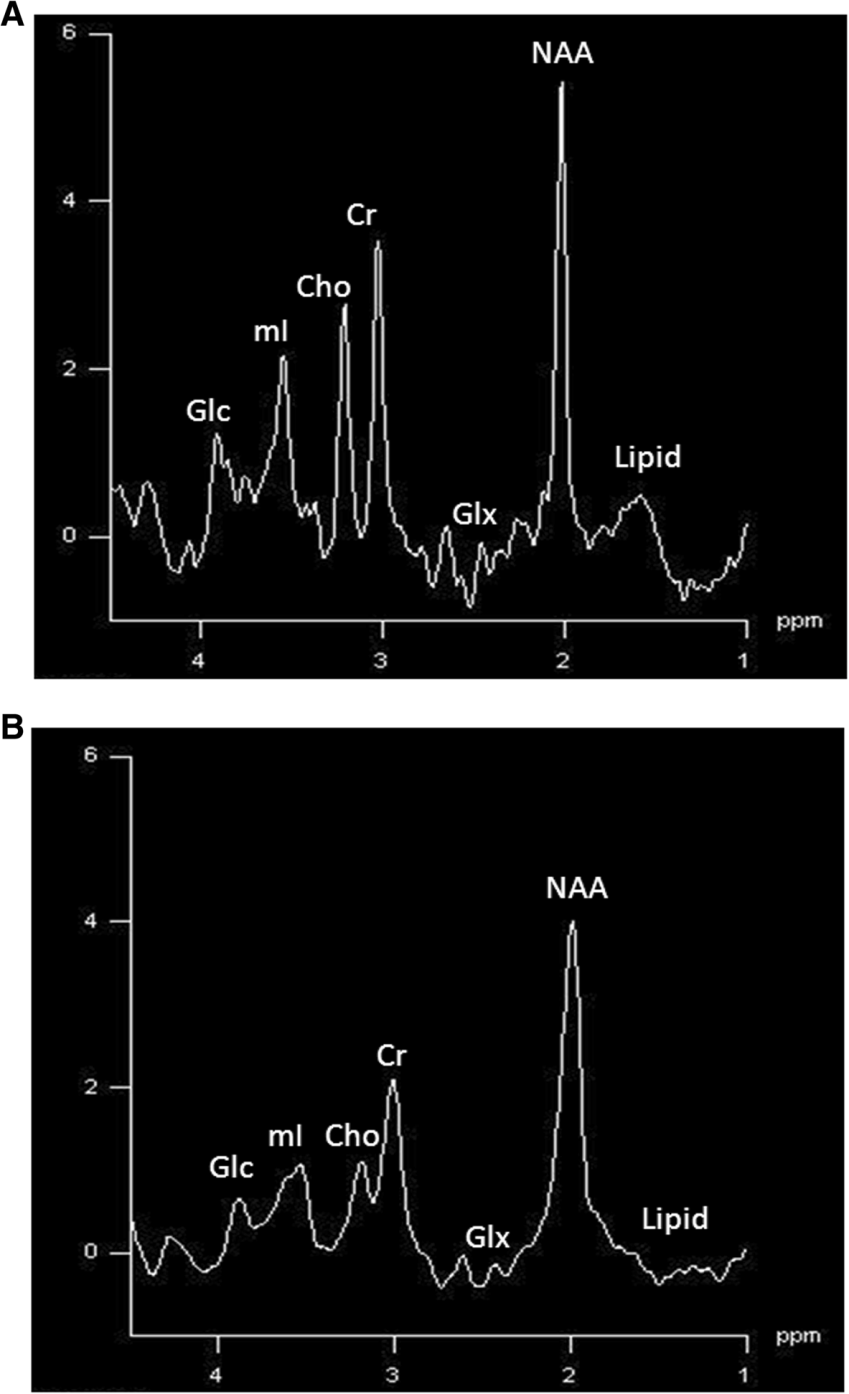
^**1**^**H-MRS (proton magnetic resonance spectrum) from right parieto-occipital region of a control (A) and a patient (B).**

The absolute concentrations of NAA, Cho, Cr, Glc and Glx, summarised in Tables 1 and 2, were calculated using LC model. The absolute concentration of NAA was found to be significantly lower in diabetic group as compared to control group in right frontal [4.35 ±0.69 vs. 5.23 ±0.74; *p* = 0.03] and right parieto-occipital region [5.44 ±0.52 vs.6.08 ±0.25; P = 0.0]. On the other hand, the level of Glx (Glu + Gln) was found significantly increased [7.98 ±2.57 vs.5.32 ±1.43; *p* = 0.01] in the right frontal area in diabetic group; however, no significant differences were observed in the other two areas of the brain examined (*p* > 0.05). Interestingly, Glc was found to be significantly higher in the three regions of the brain in diabetic group as compared to control group (*p* < 0.01). However; the absolute concentrations of Cho, mI and Cr did not show any significant difference between the two groups in the three regions of the brain under investigation (*p* > 0.05) Figure 1.

**Table 1:** Baseline characteristic of study subjects

**Parameter**	**Group1: diabetes (n = 10)**	**Group2: control (n = 7)**	***p- *** **value**
Age (yr)	39 ±5.19	27.57 ±2.76	0.19
BMI (kg/m^2^)	25.91 ±5.24	24.26 ±3.47	0.35
Serum cholesterol (mg/dl)	191 (125–239)	185.5 (124–250)	0.91
Serum LDL cholesterol (mg/dl)	123 (55–150)	123 (78–149)	1.00
Triglyceride (TG)(mg/dl)	151 (110–272)	138 (89–190)	0.66
Serum HDL cholesterol (mg/dl)	44.5(43–46)	42.5(39–50)	0.08
Systolic blood pressure (mm Hg)	131.75 ±20.7	116.66 ±8.16	0.30
Diastolic blood pressure (mm Hg)	77.75 ±2.06	76.66 ±5.16	0.15
Age at onset of DM (yr)	37.75 ±2.06	-	-
Duration of DM (yr)	3.5 ±1.2	-	-
Fasting blood glucose (mg/dl)	195.5 (113–256)	92 (87–97)	0.01
HbAIC (%)	7.25 (6.8–8.9)	5.6 (5.4–6)	0.01
CRP (Median, range)	4.8 (4.5–5.5)	0.45 (0.1–2)	0.01
Serum Insulin	6.95 (5.8–12)	-	-

**Table 2:** **Metabolite Concentration** (**mM**/**L**) **in patients with diabetes and controls**

**Metabolite ratio**	**Right frontal**		***p-*** **value (a-b)**	**Right parieto-temporal**		***p-*** **value (c-d)**	**Right parieto-occipital**		***p-*** **value (e-f)**
	**Patient (a) (n = 10)**	**Control (b) (n = 7)**		**Patient (c) (n = 9)**	**Control (d) (n = 6)**		**Patient (e) (n = 9)**	**Control (f) (n = 4)**	
NAA	4.53 ±0.69	5.23 ±0.74	0.03	5.53 ±0.42	5.83 ±0.76	0.17	5.44 ±0.52	6.08 ±0.25	0.02
Cr + PCr	3.23 ±0.26	3.43 ±0.50	0.15	3.84 ±0.31	3.61 ±0.42	0.11	3.72 ±0.26	3.86 ±0.22	0.19
GPC + PC	0.94 ±0.16	0.86 ±0.14	0.14	1.03 ±0.30	1.09 ±0.13	0.24	0.97 ±0.13	0.90 ±0.19	0.25
Glu + Gln	7.98 ±2.57	5.32 ±1.43	0.01	6.74 ±1.30	6.72 ±1.94	0.48	7.49 ±1.64	6.44 ±1.68	0.15
Glc	1.57 ±0.98	0.43 ±0.21	0.004	2.33 ±0.86	0.69 ±0.88	0.005	1.78 ±0.67	0.79 ±0.45	0.01
mI	2.93 ±0.60	2.65 ±0.43	0.15	3.06 ±0.62	2.66 ±0.36	0.09	3.03 ±0.38	2.81 ±0.25	0.16

## Discussion

In this study, the localized ^1^H-MRS was used to measure the absolute concentrations of brain metabolites in white matter areas of right frontal, right parieto-temporal and right parieto-occipital regions of normotensive type-2 DM patients and healthy controls. These data indicate that the variations in brain metabolites were not uniform across different regions of the brain studied. NAA was found significantly decreased in the right frontal and right parieto-occipital regions, while Glx showed a significant increase in frontal area in the diabetic group as compared to the control. The level of glucose was increased in all the three regions of brain in the diabetic group as compared to the control (*p* < 0.05). There were no measurable changes in the levels of Cho, Cr and mI in any of the three regions of the brain. Further, the data indicated that the frontal white matter and parieto-occipital lobes were the most affected in diabetics. Similar results were reported by Ajilore et al. and Sahin et al [4,17]. These findings are consistent with recent studies reporting that patients with type-2 DM execute persistently worse in all cognitive functions, especially tasks that involve intellectual functioning, verbal memory and psychomotor performances [7]. In addition, some studies demonstrated the involvement of white matter tract in the frontal lobe, temporal lobe, cingulate, occipital lobe and cerebral peduncle which explains the cognitive slow down in these patients [6].

Our study indicated that NAA levels were significantly decreased in diabetics as compared to the control group. NAA is an amino acid that is exclusively synthesised and localised in neurons. It acts as a major osmolyte and also serves as a marker of neuronal viability [18]. The primary role of NAA is neuronal-glial cell specific signalling for the optimal functions of the central nervous system [19]. The decline in brain NAA reflects axonal or neuronal dysfunction, or loss in neuronal density similar to that observed in stroke, hypoxia, neoplasm, epilepsy, multiple sclerosis and dementia [20]. Muranyi et al. reported that, in an animal model, hyperglycaemia causes mitochondrial swelling consequently initiating hypoxic cell death cascade in brain [21]. In our study, decreased levels of NAA observed in diabetic patients may reflect neuronal loss possibly due to chronic hyperglycaemia or ischemia. Sahin et al. studied a group of 25 patients with type-2 DM that included 13 patients with impaired glucose tolerance test and 14 healthy controls. They found that Cho/Cr ratios were increased in frontal cortex in patients with IGT where as mI/Cr ratios were increased in frontal cortex, and Cho/Cr ratios decreased in parietal cortex in patients with type-2 DM as compared to the controls. They also found that HbA1C level was inversely correlated with NAA/Cr and Cho/Cr ratios in the frontal cortex and with Cho/Cr ratio in parietal white matter [17]. However, contrary to these studies, Tiehuis et al. studied 72 patients with type-2 DM and 40 control subjects using 1H-MRS and reported no differences in NAA/Cr, Cho/Cr or NAA/Cho ratios between patients with type-2 DM and controls; and neither cognitive performance in patients with type-2 DM was correlated with any of these brain metabolites nor were the clinical variables [22]. Some authors have used ^1^H-MRS to evaluate brain metabolites in type-2 diabetic patients with other co-morbidity such as depression and hypothyroidism and have reported variable results. Ajilore et al. found, in a study comprising patients with type-2 DM with major depression (n = 24), DM alone (n = 24) and healthy controls (n = 21), significant increase in absolute concentrations of mI in frontal white matter in DM alone and DM with major depression patients as compared to healthy controls [4]. However, the study of ten patients with type-1 DM reported by Makimattila et al. showed increased levels of Cho and mI in white matter in comparison with ten healthy controls, with inverse correlation of glycaemic exposure with NAA and Cho in white matter and with Cho in grey matter [19].

The present results showed no significant differences in the concentration of Cr and mI between patients with DM and controls. Similar results were obtained by Makimattila et al. [19]. Few studies have reported significant increase in mI in frontal white matter in patients with DM with or without depression as compared to healthy subjects [4,23]. Myo-Inositol, a naturally occurring sugar and membrane constituent, reflects proliferation or activation of glia. It also serves as a cell messenger, astrocyte marker in diabetes, as well as a possible marker for intracellular osmotic integrity. Decreased levels of myo-inositol have been reported in hyperosmolar state, stroke, gliosis, tumours, lymphoma and some low grade lymphoma [24,25]. Similarly in the present study, no significant differences in the concentrations of Cho were observed between the two groups. Choline containing compounds are indicator of cell density and cell wall turnover. They also reflect metabolism of myelin and other phospholipids of cell membrane. The levels of choline containing compounds elevate under certain conditions such as in chronic ischemia, tumours, especially malignant ones, and in certain demyelinating diseases [25-27].

The present study further indicates significantly higher levels of Glu and Gln (Glx) in right frontal regions of the brain of patients with DM. Glu and Gln (Glx) are putative osmolytes and their presence may indicate fluid imbalance resulting from regular disruption of glucose homeostasis. Our results also showed significantly higher levels of glucose in all the three regions of the brain examined in DM group as compared to the control. The level of glucose may also increase during osmotic disturbances related to hyperglycaemia in DM patients. This fact is supported by the earlier studies of Makimatilla et al. and Brand et al. [19,28]. Similarly, a previous study by de Graaf et al. has shown that glucose content of the brain is linearly related to the plasma glucose level [29]. However, a recent study by Kim et al. in patients with well controlled type-1 DM showed that brain glucose content and kinetics of brain glucose transport do not differ between healthy subjects and patients with uncomplicated type-1 DM under hypoglycaemic conditions [30].

However, besides the results presented herein, this study has some limitations which need to be addressed such as small sample size. In addition, some blood tests were not done in few patients due to discontinued follow up. In conclusion, it is clear that type-2 DM causes subtle cerebral metabolic changes. Decreased NAA might be indicative of decreased neuronal viability in diabetics as a result of chronic hypoxia and hyperglycaemia. Increased glutamate and glutamine levels may be due to the effect of hypoxia and hypoglycaemia. However, these conclusions need to be confirmed by further studies with large sample size.

## Conclusions

^1^H-MRS analysis indicates that type-2 diabetes mellitus may cause subtle changes in the metabolic profile of the brain. In conclusion, it is clear that type-2 DM causes subtle cerebral metabolic changes. Decreased NAA might be indicative of decreased neuronal viability in diabetics as a result of chronic hypoxia and hyperglycaemia. Increased glutamate and glutamine levels may be due to the effect of hypoxia and hypoglycaemia. However, these conclusions need to be confirmed by further studies with large sample size.

### Availability of supporting data

The authors declare that they have no supporting data available in an open access repository.

## Competing interests

The authors declare that they have no competing interest and have not received any financial assistance for this project.

## Authors’ contributions

SS provided inputs to the study design, helped in data analysis and interpretation, wrote the manuscript, and did final editing. ME and RP reviewed literature, and helped in interpreting data and in writing the manuscript. US and NRJ did MRS of brain and collected the MRS data, reviewed literature, and helped in interpreting data and writing the manuscript. RMP did data analysis. All authors approved and read the final manuscript.
